# Account of the Efficacy of Yeast in Typhus Fever

**Published:** 1807-06

**Authors:** T. Watkins

**Affiliations:** Havre-de-Grace


### *Account of the Efficacy of* YEAST *in* TYPHUS FEVER. *By Mr.* T. WATKINS, *of Havre-de-Grace.*

AFTER reading the observations of Dr. Thornton and the Rev. Mr. Townsend on the efficacy of yeast in the cure of typhus fever, I had determined to make trial of it the first opportunity which should present itself, and am highly gratified by the result. On the 12th inst. I was called to a man, about 40 years of age, labouring under a bilious autumnal fever; he lay in a motionless insensible state, his pulse ninety, weak and intermitting; his eyes open and fixed, except on the near approach of any object, when they discovered that tremulous motion, which is customary; his mouth half open, gave me an opportunitity of inspecting his tongue and fauces, which were covered with a dark-coloured slimy bilious matter; his extremities were cold, and his whole body covered with a cold clammy sweat; now and then a trembling and twitching of the tendons of the arms and hands were observable; all attempts tempts to make him speak were ineffectual. He had been fifteen days ill, and twenty-four hours in his present situation. I immediately ordered two large blisters to be applied to his legs, and directed half a wine glassful of Maderia wine and water to be poured down his throat every two or three hours, and in the intervals tinct. cinchonæ in small quantities conjoined with aromatic tincture; for ten hours he was without the smallest perceptible alteration, but continued in the same state of insensibility, except when raised to take his wine, which he greedily swallowed when put to his mouth; in twenty-four hours the blisters were cut and dressed without rousing him. In despair of being able to save him, I ordered his mouth and fauces to be well washed with vinegar and honey, and a table-spoonful of yest to be given every three hours; in ten hours after his taking it, when his blisters were again dressed, he complained of their soreness, and spoke of his approaching death, but still appeared not to observe my entrance, and could not answer when spoken to; his pulse was slower and fuller than before, his skin warm, and his eyes had lost a good deal of their vacant stare: he had taken during the day four table-spoonfuls of yest, and drank a pint of Madeira wine; he was ordered an anodyne at night, which procured him a good sleep, and the next morning I found him sitting up in bed; his pulse was now about seventy, full, soft, and regular, and no symptom of disease remained but debility. He ate this morning a pint of panada, the first food he had taken for three days. As nothing was now necessary but to support the vital energy, I directed a continuance of the tinct. cinchonæ with wine and a generous diet, and that his bowels should be kept moderately open with some gentle medicine as senna, &c.; he continued to mend rapidly, and in three days could walk abroad.

*Sept.* 25, 1804.

